# Can Skin Allograft Occasionally Act as a Permanent Coverage in Deep Burns? A Pilot Study 

**Published:** 2017-01

**Authors:** Ezzatollah Rezaei, Arash Beiraghi-Toosi, Ali Ahmadabadi, Seyed Hassan Tavousi, Arash Alipour Tabrizi, Kazem Fotuhi, Mehdi Jabbari Nooghabi, Amir Manafi, Shokoofeh Ahmadi Moghadam

**Affiliations:** 1 Surgical Oncology Research Center, Mashhad University of Medical Sciences, Mashhad, Iran;; 2 Iranian Legal Medicine Research Center, Legal Medicine Organization, Tehran, Iran;; 3 Department of Surgery, Imam Khomeini Hospital, Northern Khorasan University of Medical Sciences, Shirvan, Iran;; 4 Shahid Beheshti University of Medical Sciences, Tehran, Iran;; 5 School of Medicine, Islamic Azad University, Tehran Branch, Tehran, Iran

**Keywords:** Skin allograft, Burn, Polymerase chain reaction

## Abstract

**BACKGROUND:**

Skin allograft is the gold standard of wound coverage in patients with extensive burns; however, it is considered as a temporary wound coverage and rejection of the skin allograft is considered inevitable. In our study, skin allograft as a permanent coverage in deep burns is evaluated.

**METHODS:**

Skin allograft survival was assessed in 38 patients from March 2009 to March 2014, retrospectively. Because of the lack of tissue specimen from the skin donors, patients with long skin allograft survival in whom the gender of donor and recipient of allograft was the same were excluded. Seven cases with skin allograft longevity and opposite gender in donor and recipient were finally enrolled. A polymerase chain reaction (PCR) test on the biopsy specimen from recipients and donors were undertaken.

**RESULTS:**

PCR on the biopsy specimen from recipients confirmed those specimens belong to the donors. All patients received allograft from the opposite sex. Two (28.57%) patients received allograft from their first-degree blood relatives, and in one (14.29%) case, the allograft was harvested from an alive individual with no blood relation. The rest were harvested from multiorgan donors. In eight months of follow up, no clinical evidence of graft rejection was noted.

**CONCLUSION:**

Long term persistence of skin allograft in patients is worthy of more attention. Further studies An increase in knowledge of factors influencing this longevity could realize the dream of burn surgeons to achieve a permanent coverage other than autograft for major burn patients.

## INTRODUCTION

Skin allograft is considered to be the gold standard for the treatment of burns in patients who do not have enough skin to cover all the burned parts of the body.^1^ However, it is regarded a temporary coverage and many studies demonstrated that if allograft is not replaced by autograft, graft rejection is likely to occurwithin two weeks.^2^^,^^3^ This urged burn surgeons to find a replacement for the patients’ own skin, especially for those with susbstantial burns. As an attempt to solve such a problem, some investigators successfully used human leukocyte antigen (HLA)-matched allografts.^3^

However, this is not always a possible solution due to the limited number of allograft donors. Transplanted allograft skin is more prone to graft rejection compared to transplant allograft kidney and heart; in fact, the only organs prone to graft rejection similar to the skin allograft are the small intestine and the lungs.^4^ Many investigators have attempted to find a way to postpone the graft rejection process. The methods used to prolong allograft survival can be divided into two categories: First are the methods trying to reduce the antigenicity of allograft before the transplant surgery and delay the graft rejection. Second are the methods trying to achieve the same goal by modifying the response of the immune system of the skin allograft recipient.^5^

Obviously, it is due to the vulnerability of burn patients to infections that immunomodulation of the allograft tissue is a more proper and easier solution to prevailearly rejection than immunosuppression of the patient. However, even without allograft antigenicity reduction techniques and immune-suppressive drugs, sometimes it may be observed that allograft persists for a long time and with rarity, it could permanently act as the patient’s skin, showing no evidence of graft rejection.^5^ Burn is still as the most devastating condition in emergency medicine in developing and developed countries, leading to physical and psychological scars and disabilities.^6^ So this study was undertaken to evaluate skin allograft as a permanent coverage in deep burns.

## MATERIALS AND METHODS

Because of the lack of skin bank in Iran, fresh skin allograft transplantation was started at the Department of Burns and Reconstructive Surgery of Imam Reza Hospital in Mashhad, Iran, from 2004. Skin allograft in this center is supplied from two main resources: First is the skin allografts of the brain dead multi organ donors and the other is the split thickness skin harvested from the patients’ relatives. In the former cases, all of the skin allografts were used in less than 24 hours and in the latter cases, this time period was less than 2 hours. Due to the limited availability of allografts, all the allografts (except for the allografts used in the face and hands) are meshed in a ratio of 1:1.5or 1:3.

This study was approved by ethics committee of Mashhad University of Medical Sciences. We reviewed results of skin allograft longevity in burn patients who were admitted in the burns department. We extracted records related to skin allograft transplantation from Hospital Information System from March 2009 to March 2014 (38 cases). Then we exclude records related to the expired patients (5 patients). Because of the lack of the tissue specimen from skin allograft donors, we compared gender of donors and recipients and excluded cases with same gender of donor and recipient (14 cases). 

There were 19 alive cases with opposite donor and recipient genders. In twelve of them, skin allograft had been rejected and patients need to skin autograft who were excluded from the study. Finally, we had seven cases with skin allograft survival up to this time. We obtained skin biopsy from site of the tissue transplantation and studied specimens by polymerase chain reaction (PCR) test to find sex chromosomes related to opposite gender in recipients.

## RESULTS

**Fig. 1:**
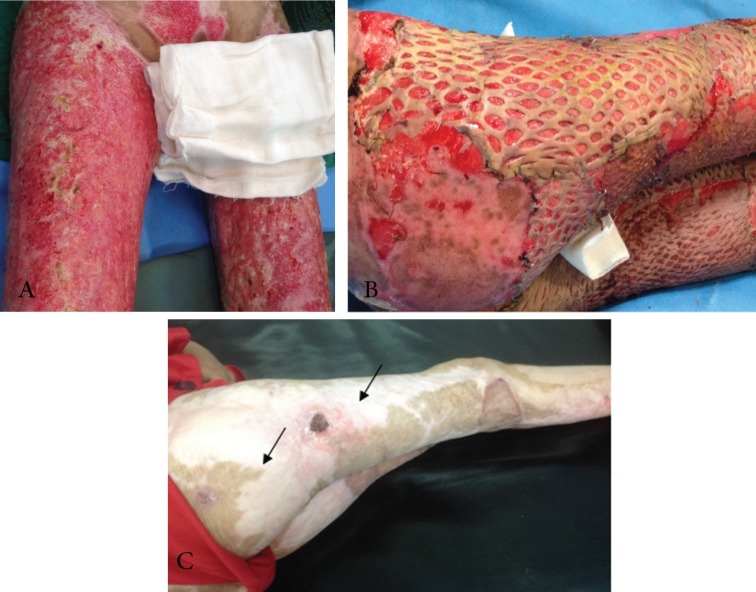
(a) Image of burns in a 12-year-old girl; view of wounds 53 days after injury confirms deep burns in this patient. (b) Four days after skin allograft transplantation. (c) 255 days after burns accident. Extensive depigmented skin in areas covered by skin allograft is remarkable. Small areas of wound bed witch has not been covered by skin allograft (the arrows) are not yet repaired after 10 months than burn accident

**Fig. 2:**
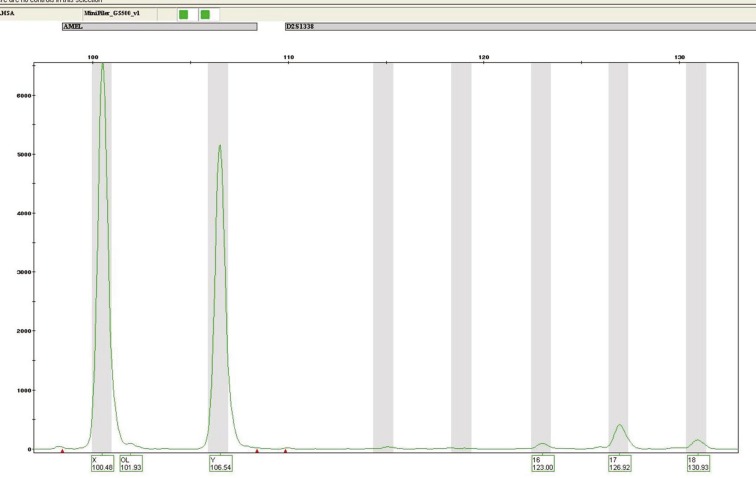
A polymerase chain reaction test on a biopsy specimen from a 6-month baby girl who received allograft from her father

Among 38 patients, 7 patients were enrolled in the study. The mean age of the patients was 12.57±10.36 years (6 months to 28 years). Six (85.71%) patients were female and one (14.29%) was male. All patients received allograft from the opposite sex. Two (28.57%) patients received allograft from their first-degree blood relatives, and in one (14.29%) case, the allograft was harvested from an alive individual with no blood relation. The rest were harvested from multiorgan donors. The characteristics of donors and recipients, and the survival time of the allograft were shown in Table 1. Skin allograft survival time from transplantation to biopsy obtaining was between 43 and 1797 (510.14 ± 613.55) days (Figure 1). In eight months of follow up from last biopsy obtaining to date, we observed no clinical evidence of graft rejection (Figure 2).

**Table 1:** The characteristics of the donors and recipients and the survival time of allograft

**Cases**	**Age (year)**		**Sex**		**Preservation time (hrs)**	**Burnt TBSA** * **(%)**	**Allograft survival time (days)**	**Source of allograft**
	**Donor**	**Recipient**	**Donor**	**Recipient**				
1	41	22	Male	Female	21	70	1797	Cadaver
2	19	3.5	Male	Female	19	35	628	Cadaver
3	33	18	Male	Female	24	52	599	Cadaver
4	40	11	Male	Female	17	45	255	Cadaver
5	35	28	Female	Male	1	60	132	Alive
6	27	5	Male	Female	1	45	117	Alive
7	39	0.5	Male	Female	1	20	43	Alive
Mean (SD)	33.43 (7.99)	12.57 (10.36)	Male % =85.71	Male % =14.29	12.00 (10.50)	46.71 (16.35)	510.14 (613.55)	Alive % =42.86

* Total body surface area

## DISCUSSION

Human skin allograft has been probably used in wound coverage for as long a time as the autogenic skin transplantation. In contrary to the fact that allogenic skin had been considered as a permanent wound coverage up to 1914, at this time the incompatibility of cadaver skin was firstly described by Lexer.^7^ It is predominantly believed that xenografts and allografts only provide a temporary coverage in burn wounds. Only in cases of living autografts or isografts, i.e., identical twins, the true closure can be achieved.^8^

Many investigators attempted to prolong skin allograft survival by reducing allograft antigenicity or suppressing immune system of the patients. One of the most common methods for reducing the antigenicity of allograft skin is using glycerol to preserve the allograft.^5^ The reason allograft antigenicity decreases while preserved in glycerol is that the living structures in the skin are destroyed by the glycerol, that is why it is not considered a living tissue.^7^^,^^8^

In addition, Glycerol has antibacterial and virus-killing properties.^5^^,^^9^^,^^10^ It has been also discovered that cryopreservation of the skin allograft at -196°^C^ causes the Langerhans cells degeneration, which prolongs viability of cryopreserved allograft.^11^ Researchers found that the number of mesenchymal origin cells, including melanocytes, is less than normal in cryopreserved skin.^11^^,^^12^

When the integration of the allograft dermal layer is desired, the usage of cryopreserved skin allograft is preferred.^7^ As an alternative, the use of ultraviolet B radiation and steroids before transplantation of skin allograft can increase skin allograft survival.^13^ Azathioprine, antithymocyte globulin, steroids and cyclosporine have been used to prolong the skin allograft survival.^14^ In another study, Cyclosporine was applied to prolong skin allograft survival, which successfully saved the life of the patient without any side effects related to cyclosporine.^15^

However, permanent persistence of allograft without immunosuppression has been reported and it is presumed to be due to incidental close HLA matching, burn induced immunosuppression, or gradual substitution of allograft with autologous epithelium. Although, in case of wide and deep burns where there is no autogenous epidermis on the wide surface, such events seem to be rather far-fetching.^3^

Phipps and Clark reported on their experience in the use of intermingled autograft and parental allograft skin in the treatment of major burns in 10 children that among 10 children, 3 patients achieved complete healing, and 2 others required only autografts. They did not find any histological evidences of acute rejection in biopsy specimens; but in the only one case which they used Y-probe, they were not able to demonstrate the survival of male cells in their female patient.^3^ Nonetheless, it is noteworthy that using Y-probe method is not error-free and its authenticity is hardly comparable with our study in which the PCR method was applied.

In another study, Qaryoute *et al.* provided a report on their experience in the use of widely meshed autologous skin, overlaid with meshed allograft from a parent of five children. Their clinical and in some cases histological observations demonstrated that the parental skin persisted for a long time.^2^ We do not know the exact cause of long-term allograft survival in patients. However, given how we use allograft, several hypotheses have attempted to explain the long-term survival of allograft.

To begin with, unlike many other studies, we used fresh allograft and all the allografts were preserved for less than 24 hours. It could be assumed that the longer the allograft preserved, the more ischemia is imposed to its cells. Subsequently, the risk of inflammatory reaction and graft rejection would be increased.

Castagnoli *et al. *evaluated cell viability index in the fresh and cryopreserved skin in 350 samples harvested from 35 donors. In their study, samples tested 12–30 hours after harvesting had an average viability index of about 75 and samples tested within 60 hours from harvesting had an average viability index of 40, showing a viability decrease of about 50%.^16^

The second hypothesis considers it likely that because all the donors and recipients of the allografts were from the same geographical population, there is the probability of high genetic similarity among them. On the other hand, in many cases where the allograft has persisted, the skin in much of the area covered with allograft is amelanotic. This finding may suggest that in these patients, dermal mesenchymal cells such as the langerhans cells and melanocytes, which bear more antigenic properties, have been demolished and those cells with less antigenic properties such as the keratinocytes have remained. approving or rejecting each of these hypotheses requires further studies.^11^^,^^12^

In conclusion, long term persistence of skin allograft in some patients is a fact which is worthy of more attention. Further studies are recommended to investigate the genetic similarity of the skin allograft donors and recipients. An increase in knowledge of factors influencing this longevity could realize the dream of burn surgeons to achieve a permanent coverage other than autograft for major burn patients.
